# Multicompartmental Pharmacokinetic Model of Tenofovir Delivery to the Rectal Mucosa by an Enema

**DOI:** 10.1371/journal.pone.0167696

**Published:** 2017-01-23

**Authors:** Yajing Gao, David F. Katz

**Affiliations:** 1 Department of Biomedical Engineering, Duke University, Durham, NC, United States of America; 2 Department of Obstetrics and Gynecology, Duke University Medical Center, Durham, NC, United States of America; Harvard Medical School, UNITED STATES

## Abstract

Rectal enemas that contain prophylactic levels of anti-HIV microbicides such as tenofovir have emerged as a promising dosage form to prevent sexually transmitted HIV infections. The enema vehicle is promising due to its likely ability to deliver a large amount of drug along the length of the rectal canal. Computational models of microbicide drug delivery by enemas can help their design process by determining key factors governing drug transport and, more specifically, the time history and degree of protection. They can also inform interpretations of experimental pharmacokinetic measures such as drug concentrations in biopsies. The present work begins rectal microbicide PK modeling, for enema vehicles. Results here show that a paramount factor in drug transport is the time of enema retention; direct connectivity between enema fluid and the fluid within rectal crypts is also important. Computations of the percentage of stromal volume protected by a single enema dose indicate that even with only a minute of enema retention, protection of 100% can be achieved after around 14 minutes post dose. Concentrations in biopsies are dependent on biopsy thickness; and control and/or knowledge of thickness could improve accuracy and decrease variability in biopsy measurements. Results here provide evidence that enemas are a promising dosage form for rectal microbicide delivery, and offer insights into their rational design.

## Introduction

Topically acting microbicides are being developed as an important prophylactic modality in the fight against infection by sexually transmitted HIV[1–3]. Different topical dosage forms have been proposed and are in various stages of development; these include gels, intravaginal rings, solid inserts and suppositories, and films[4–8]. Original microbicide development was for vaginal application, and was followed by rectal microbicide candidates[9]. Infection by HIV virions due to receptive anal intercourse is globally pandemic, and the need to mitigate it is substantial. Different microbicide product types have been intended for application to the vaginal vs. rectal canals. Gels were the original dosage form, originally for the vagina and later for the rectum[10]. Recently an enema vehicle has been proposed for rectal microbicide delivery[2], and is being evaluated in multidisciplinary studies. Potential advantages of this dosage form include: (1) cleansing enemas are often used by people prior to receptive anal intercourse (RAI); these would act to clear fecal contents from the rectal canal that could impede subsequent medicated enema distribution and drug delivery; further, there could be minimal change in behavior required for product application prior to sex; (2) the low viscosity of enema fluid (e.g. as compared to that of a gel) would promote good spreading and contact with the rectal mucosa for drug delivery; and (3) the relatively high enema fluid volume could deliver more drug in a shorter amount of time than would a smaller volume typical for vaginal gels.

Many factors must be taken into account in design of a microbicide enema product. Historically, such design and product development have been based primarily upon experimental studies, *in vitro* and *in vivo*. A central focus (given that product safety and stability are achieved) has been achieving target pharmacokinetics (PK) for the active pharmaceutical ingredient (API). This PK depends upon multiple factors including: drug properties (e.g. diffusion and partition mass transport coefficients in different compartments; drug solubility); vehicle properties (e.g. enema rheology and other physicochemical properties, volume, drug loading); the time interval between application of an enema and its expulsion; and properties of the rectal canal and mucosa (e.g. anatomical and histological characteristics).

Computational compartmental PK models can contribute to our understanding of how microbicide PK depends upon the many factors that deterministically govern it[11]. Deterministic PK models have been developed for vaginal microbicide gels and intravaginal rings[12] and are now being used in gel design[13–15]. Such modeling is providing valuable information about how interactions between gel properties and applied volume govern PK[12,16]. Recently, the modeling has begun to explain how details of microbicide PK translate to prophylactic efficacy against vaginal mucosal infection by HIV. Inputting data on properties of a gel and the vaginal environment, it has predicted the time interval between gel insertion and subsequent creation of prophylactic mucosal drug concentrations (within the lamina propria, viz. stroma), the degree of such protection, and its duration[12,16].

The analysis here initiates counterpart PK model development for a microbicide enema, focusing upon the drug tenofovir which (and also its prodrugs) is a leading API for a microbicide enema product. Tenofovir (TFV) is phosphorylated to tenofovir diphosphate (TFV-DP) in mucosal cells, including infectible host cells to HIV that reside in the mucosal stroma. There it acts against HIV as a nonnucleotide reverse transcriptase inhibitor. In many ways, the rectal mucosal environment is more geometrically, histologically and biophysically complex than the vaginal one. In contrast to the roughly four-fold thicker, multi-layered stratified squamous epithelium of the vagina (in humans), the rectal mucosal contains a single layer of columnar epithelial cells above the stroma and these are geometrically arranged to create a mosaic of fluid-filled crypts (often termed crypts of Lieberkühn) down into the tissue. Goblet cells in the rectal epithelium secrete mucus that coats the surface and fills the crypts. These features, which are distinct from those of the vaginal environment, can have a significant effect on drug mass transport, and should be addressed in creating and applying rectal PK models. Our approach is inherently deterministic, employing principles of mass transport relevant to each of the contiguous compartments through which tenofovir migrates, from the enema fluid to the blood stream. Consistent with the evolution of mass transport modeling in other biological systems, we begin here with a model that addresses salient factors which govern PK while applying simplifications to certain details of the host environment. These simplifications reduce the multivariate complexity of the analysis and limit reliance upon data (parameters) that may not yet be known specifically for this problem. In particular, the model focuses upon effects of: (1) varying enema retention time; (2) varying crypt geometry[17]; (3) the direct openness of the crypts to luminal fluid and drug therein[18]; and (4) varying advective fluid flow in the crypts, e.g. due to active rectal fluid absorption and/or contrasting tonicity of the enema fluid[19]. A primary simplification is assumption that there is complete, uniform distribution of enema fluid along the length of the rectum along which this analysis applies. This is a reasonable assumption for the first rectal PK model, akin to that for the initial vaginal model[20], which was followed by derivative models addressing additional factors governing microbicide PK. Here, consistent with practice by many who engage in RAI, we assume de facto that a cleansing enema has been applied before the medicated one. This would act to remove feces that could impede distribution of the medicated enema fluid. Because of the low viscosity of that fluid, its distribution along the canal would be rapid. As a result, the time scale of enema fluid transport along the canal would be fast compared to the scale of drug transport out from the enema and into the rectal mucosa. Put another way, drug transport would be inherently diffusion limited, as embodied in the model here. In the absence of a prior cleansing enema, feces would impede distribution of medicated enema fluid and reduce delivery of API to target cells. Follow up analysis should address the time-dependent, non-uniform distribution of the feces and enema fluid[21,22] as well as other physiological and anatomical factors, e.g. as related to rectal anatomy and motility. The ‘perfect distribution’ model here is a building block for more complex analysis. That will be akin to analyses we have created and applied in understanding microbicide delivery by vaginal gels[12,20].

## Methods

### Geometry of the model

**Fig 1:**
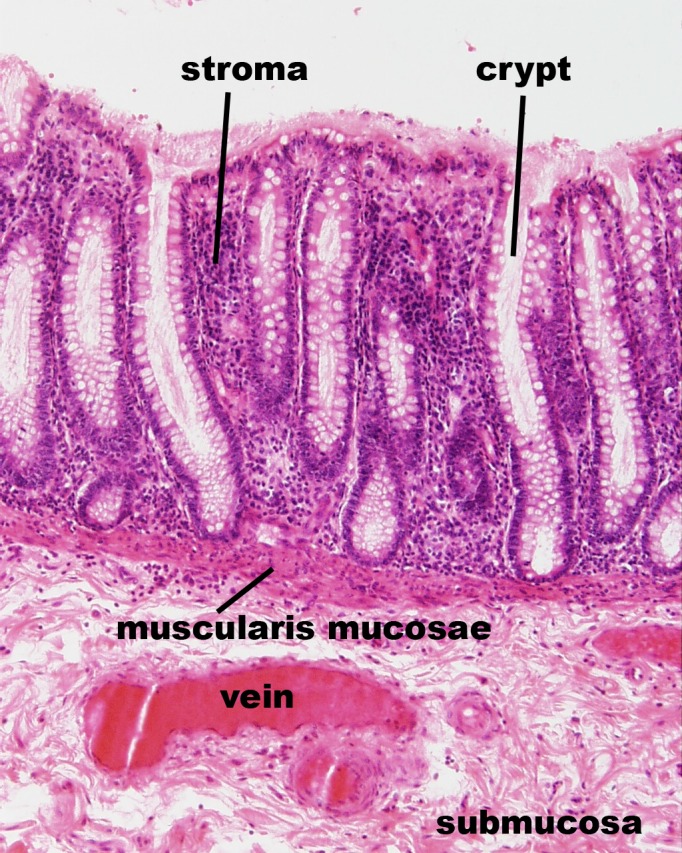
Histological image of therectal mucosa (longitudinal section). Rectal crypts with simple columnar epithelium are visible, and the stroma (lamina propria) is seen wrapping around the crypts. Reprinted from http://www.lab.anhb.uwa.edu.au/mb140/ [25].

**Fig 2:**
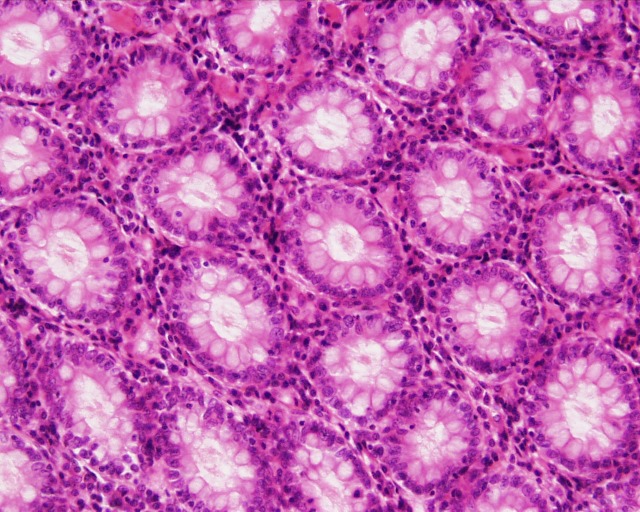
Histological image of the rectal crypts (transverse section). Regular spacing of the crypt openings is seen. Reprinted from http://www.lab.anhb.uwa.edu.au/mb140/ [25].

The geometry and morphology of the lower colorectal canal vary with location, and exhibit both macroscopic and microscopic features[17,23]. On the macroscopic scale rectal folds create creases and canyons on the mucosal surface. On the microscopic scale, there are rectal crypts and smaller crevices. The large folds are found in the relatively short anal canal, which is about 2 to 4 cm long, ending in the anal verge[24]. This contains a stratified squamous epithelium (Fig 1). The rectum possesses crypts (Fig 1 and Fig 2) on its surface with a thin columnar epithelium. These are about 40–120 μm in diameter and up to about 1 mm in depth. As seen in Fig 2, they are regularly spaced (about 150 μm apart). The characteristic length for transport into the mucosal tissue is only about one millimeter, i.e. the thickness of the mucosa. Larger features, such as curvatures of the surface and of the large folds, can be neglected (i.e. approximated as flat) due to the small characteristic drug transport length into the mucosa.

**Fig 3:**
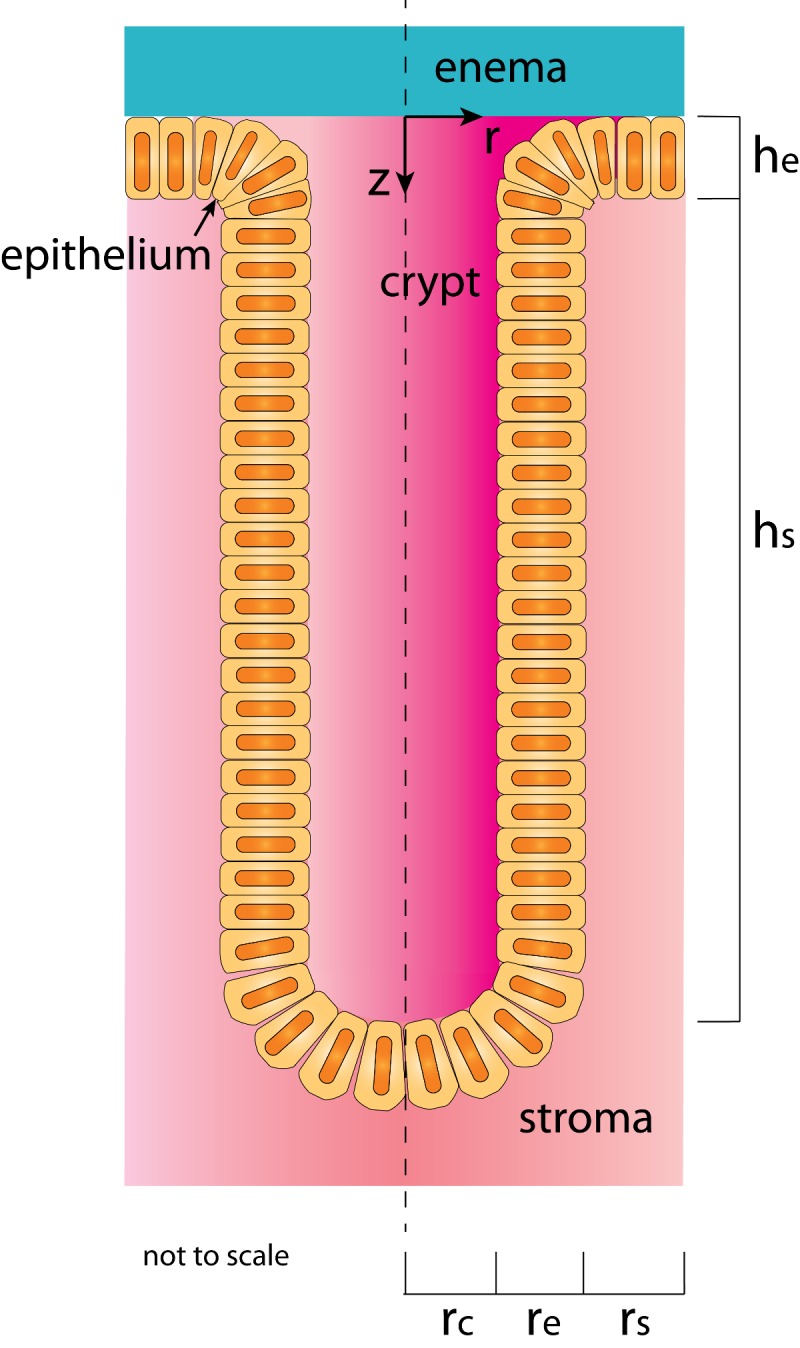
Drawing of the crypt geometry in this model. The figure is in cylindrical coordinate system used for the computations. The enema fluid fills the canal above the crypt.

We represent the geometry of the canal and the crypts as a central longitudinal circular cylinder (the canal) into which small cylinders (the crypts) insert at right angles (Fig 3). The epithelium contains a mosaic of rectal mucus-filled crypts, which can have varying sizes (see Fig 2 in which a range of crypt diameters is visible). The characteristic lengths for each crypt are its radius, the thickness of its epithelial layer, the separation distance between adjacent crypts, and the depth of the crypt down into the mucosal tissue. The enema fluid fills the canal into which the crypt inserts, covering the mucosal surface and the crypt opening. We consider two contrasting cases: “open” and “closed” crypts. For the former, there is direct contact and drug exchange between the enema fluid and the mucus within the crypt. For the latter, the apex of the crypt is closed to, and does not contact, enema fluid. The core analysis here focuses upon drug transport into a single crypt and thence into its mucosa. Total drug transport is then the summation over all the crypts. This geometry lends itself to uses of a cylindrical coordinate system within each crypt (Fig 3) with origin at the top of the crypt (z = 0). Transport is axisymmetric, and depends only upon the radial and longitudinal dimensions r and z. There are thus 5 length scales for the overall drug transport problem: *rc*, *re* and *rs* are the radial dimensions for the crypt, epithelium and stroma respectively; and *he* and *hs* are the thicknesses of the epithelium and stroma within the tissue.

### Governing equations in the compartmental model

There are five compartments overall in the model: the enema fluid within the lumen, the interior of a crypt, epithelium, stroma and the bloodstream. The luminal enema fluid compartment is taken as a reservoir (constant, uniform concentration) since the mass of drug transported from it to the crypt is a small fraction of drug mass in the fluid. The governing equations of conservation of mass for tenofovir and tenofovir diphosphate have been derived in relation to our previous work on drug delivery by a vaginal gel[12,20]. The key difference is the cylindrical coordinate system here vs. a rectangular coordinate system previously. Advective transport terms within the crypt and tissue are introduced to account for possibly varying degrees of water uptake by the rectum (see below)[19].

$$\frac{\partial C_{TFV}}{\partial t}=D_{f}\frac{1}{r}\frac{\partial }{\partial r}\left(r\frac{\partial C_{TFV}}{\partial r}\right)+D_{f}\frac{\partial^{2}C_{TFV}}{\partial z^{2}}-\nu_{c}\cdot \nabla C_{TFV}$$ (1)

$$\frac{\partial C_{TFV}}{\partial t}=D_{e}\frac{1}{r}\frac{\partial }{\partial r}\left(r\frac{\partial C_{TFV}}{\partial r}\right)+D_{e}\frac{\partial^{2}C_{TFV}}{\partial z^{2}}-\nu_{t}\cdot \nabla C_{TFV}-k_{on}\left\{C\phi_{e}-\frac{C_{DP}}{n}\right\}+k_{off}C_{DP}$$ (2)

$$\frac{\partial C_{TFV}}{\partial t}=D_{s}\frac{1}{r}\frac{\partial }{\partial r}\left(r\frac{\partial C_{TFV}}{\partial r}\right)+D_{s}\frac{\partial^{2}C_{TFV}}{\partial z^{2}}-\nu_{t}\cdot \nabla C_{TFV}-k_{b}C_{TFV}-k_{on}\left\{C\phi_{s}-\frac{C_{DP}}{n}\right\}+k_{off}C_{DP}$$ (3)

$$\frac{\partial C_{DP}}{\partial t}=k_{on}\left\{C_{TFV}\phi -\frac{C_{DP}}{n}\right\}-k_{off}C_{DP}$$ (4)

$$V_{B}\frac{dC_{TFV}}{dt}={\dot{M}}_{SB}(t)-k_{L}C_{TFV}$$ (5)

The governing conservation of mass equations for tenofovir and tenofovir diphosphate are given in Eqs 1–5. The first, Eq 1, is the transport of TFV within the crypt fluid. Here *C*_*TFV*_ is the concentration of TFV, *D*_*f*_ is the diffusion coefficient in the fluid, *ν*_*c*_ is the advective velocity down the crypt, *t* is time, *r* is the radial dimension, and *z* is the depth dimension down into the tissue. Eq 2 gives the transport of TFV in the epithelium, where *D*_*e*_ is the diffusion coefficient, *ν*_*t*_ is the advective velocity in the tissue, and *C*_*DP*_ is the local tenofovir diphosphate concentration. The last two terms account for TFV-DP production and loss kinetics: the first term gives formation of TFV-DP with a rate constant *k*_*on*_ and the second term is the elimination of TFV-DP with rate constant *k*_*off*_. The curly brackets are Macaulay brackets (a term becomes zero when it is negative inside the bracket; this ensures that the formation rate is always formally positive). Inside the Macaulay bracket *ϕ*_*e*_ is the volume fraction of cells in the epithelium; due to the close packing of cells its value is close to one. *n* is the ratio of TFV-DP to TFV inside each cell at steady state. The third equation, Eq 3, is conservation of mass for TFV in the stroma. This equation is almost identical to that for the epithelium, with *ϕ*_*s*_ as the volume fraction of cells in the stroma, and the addition of a term for loss to the bloodstream with rate *k*_*b*_. We assume that capillaries are uniformly distributed in the stroma, so that the uptake of each drug molecule by the blood is directly proportional to the local drug concentration; this leads to a first order kinetic term[20]. Eq 4 is conservation of mass for TFV-DP in the epithelium or the stroma: here *ϕ* is the volume fraction specific to each of those compartments. The advective velocities in both the crypt and the tissue are dependent on position and net volumetric advective flow to the rectum overall (see below). Finally, Eq 5 is for the homogenous blood compartment with a volume of distribution for the body *V*_*B*_, a mass transfer rate of TFV from the stroma to the blood ${\dot{M}}_{SB}$, and clearance rate constant from the blood *k*_*L*_.

Eqs 6–16 give the initial and boundary conditions.

C(z≥0,t=0)=0 (6)

$$C(z<0,t=0)=C_{0}$$ (7)

$$C(z=0,t\leq t_{e})=\Phi_{fe}C_{0}$$ (8)

$$\frac{\partial C}{\partial z}(z=0,t>t_{e})=0$$ (9)

$$C_{f}(r=r_{c})=\Phi_{fe}C_{e}(r=r_{c})$$ (10)

$$D_{f}\frac{\partial C_{f}}{\partial r}(r=r_{c})=D_{e}\frac{\partial C_{e}}{\partial r}(r=r_{c})$$ (11)

$$C_{e}(r=r_{c}+r_{e},z=h_{e})=\Phi_{es}C_{s}(r=r_{c}+r_{e},z=h_{e})$$ (12)

$$D_{e}\frac{\partial C_{e}}{\partial r}(r=r_{c}+r_{e})=D_{s}\frac{\partial C_{s}}{\partial r}(r=r_{c}+r_{e})$$ (13)

$$D_{e}\frac{\partial C_{e}}{\partial z}(z=h_{e})=D_{s}\frac{\partial C_{s}}{\partial z}(z=h_{e})$$ (14)

$$\frac{\partial C}{\partial r}(r=r_{c}+r_{e}+r_{s})=0$$ (15)

$$\frac{\partial C}{\partial z}(z=h_{e}+h_{s})=0$$ (16)

The initial condition within the crypt is zero concentration (Eq 6), and the initial condition within the luminal fluid is constant concentration, C_0_ (Eq 7). The time-dependent boundary conditions at the top crypt surface are given by Eq 8 and Eq 9. The boundary condition for TFV concentration at the surface is given by enema concentration *C*_*0*_ multiplied by the partition coefficient between the fluid and the epithelium Φ_*fe*_. For a closed crypt, the boundary condition at the top surface of the crypt is no flux, both during enema retention and after expulsion. At the interface between the crypt fluid and epithelium, there are two boundary conditions: one is on TFV concentration, with the partition coefficient Φ_*fe*_ (Eq 10); and the other is continuous TFV flux (Eq 11). Analogous boundary conditions occur at the interface between the epithelium and the stroma: here with partition coefficient Φ_*es*_ (Eq 12), and also continuous flux conditions (Eqs 13 and 14). The boundary condition in the radial direction at the outer margin between the stromas of adjacent crypts is assumed to be no flux due to symmetry (Eq 15), and the boundary condition at the bottom of the crypt tissue is also no flux (Eq 16).

### Parameters in the model

These are given in Table 1. The crypt may be filled with mucus, and we assume that the diffusion coefficient for TFV within this mucus is similar to that which we measured for the clinical TFV gel which has comparable hydration[26]. Many parameters are the same as in our previous analysis for a vaginal gel[12]. The volume of enema is applied at 125 mL[27] with a concentration of 1x10^7^ ng/mL (a 1% solution) and this is well within solubility limit for tenofovir. The diffusion coefficient[28] for the epithelium is 1x10^-7^ cm^2^/s and for the stroma is 4x10^-7^ cm^2^/s. Due to the hydrophilic nature of tenofovir, the partition coefficient of the drug is taken as 0.75 between the fluid and epithelium[28], and is unity at the interface between epithelium and stroma. Parameters relating to crypt morphometry are taken from measurements of histological images. The crypt is allowed to be of two different sizes—large or small (Fig 2). The large crypt has a radius of 60 μm, and a stromal separation distance of 45 μm to the next crypt. The small crypt has radius of 20 μm for radius and the same stromal separation distance. Epithelial and stromal thicknesses are 15 μm and 1000 μm respectively. The diameter of the rectum is around 2 cm[29]. The total retention time of the enema is taken to be 5, 10, or 20 minutes. The rate constant for the transport of TFV to blood, volume of distribution, and the clearance rate are taken as the value obtained in our vaginal gel model[20]. It is not yet possible to directly estimate advective fluid velocities, as discussed above. In the model here we considered an upper bound for advective velocity based upon measurements of net fluid absorption by the rectum[19]. The maximum advective velocity was estimated by dividing the highest rate of measured water uptake[19] by the total surface area of the rectum[19]. Parameters related to TFV-DP formation kinetics include the volume fractions of cells in the epithelium and stroma, rates of formation and elimination of TFV-DP, and the steady state ratio of TFV-DP to TFV. In our vaginal model these rates were estimated from pharmacokinetic studies of diphosphate formation [30].

**Table 1:** Parameters in the model.

Parameter	Symbol	Value
Enema TFV concentration	*C*_*0*_	1x10^7^ ng/mL
Enema volume	*V*	125 mL
Diffusion coefficient in fluid	*D*_*f*_	6x10^-6^ cm^2^/s
Diffusion coefficient in epithelium	*D*_*e*_	1x10^-7^ cm^2^/s
Diffusion coefficient in stroma	*D*_*s*_	4x10^-7^ cm^2^/s
Partition coefficient of fluid/epithelium	*Φ*_*fe*_	0.75
Partition coefficient of epithelium/stroma	*Φ*_*es*_	1
Depth of crypt	*L*	1 mm
Radius of crypt (large/small)	*r*_*c*_	60 μm, 20 μm
Stromal radii (large/small)	*r*_*s*_	45 μm, 20 μm
Epithelial thickness	*r*_*e*_,*h*_*e*_	15 μm
Stromal thickness	*h*_*s*_	1000 μm
Diameter of the rectum	*D*_*r*_	2 cm
Time of enema application	*t*_*e*_	5 min, 10 min, 20 min
Rate constant of transport to blood	*k*_*b*_	0.119 hr^-1^
Volume of distribution	*V*_*B*_	75 L
Clearance rate from blood	*k*_*L*_	1.41 hr^-1^
Advective velocity in crypt	*v*_*c*_	computed
Advective velocity in tissue (max)	*v*_*t*_	0.52 μm/s
Volume fraction of cells in epithelium	*φ*_*e*_	0.95
Volume fraction of cells in stroma	*φ*_*s*_	0.1
Rate of formation of TFV-DP	*k*_*on*_	0.693 hr^-1^
Rate of elimination of TFV-DP	*k*_*off*_	0.00413 hr^-1^
Equilibrium ratio of TFV-DP to TFV	*n*	0.1

### Solution of governing equations

Numerical solution for the system of differential equations in Eqs 1–5, subject to the boundary and initial conditions in Eq 6–16, was obtained using a finite difference method. The physical space was first discretized in the radial and depth directions with 5 micron spacing. Spatial derivatives were computed using the central difference method. The result was a system of coupled ordinary differential equations at each time point, which was then solved using Matlab’s[31] Runge-Kutta 4,5 method. Compared to the two-dimensional vaginal gel problem in a rectangular coordinate system, special attention was needed to adapt to the two-dimensional enema problem in cylindrical coordinates. Second derivatives involving the radial distance r were taken at a half step between each point, and volume integrals for calculating spatial average concentrations were evaluated specifically in cylindrical coordinates.

## Results

### Solution of model

**Fig 4:**
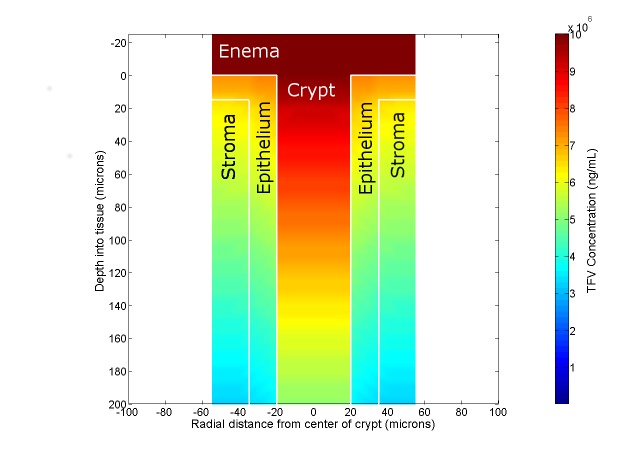
Heat map plot of tenofovir concentration distribution. The region is in the upper portion of a crypt and the adjacent mucosal tissue at 5 minutes post enema insertion. The crypt has a small size (cf. Table 1) and is open to the enema fluid (see text).

The fundamental solutions to this model are the concentration distributions of tenofovir and tenofovir diphosphate as functions of position and time after enema application within the crypt fluid and local mucosal layers, and the volume average concentration in the blood. Because of the axial symmetry in the model (see Fig 3), the spatial dependence is in two dimensions (radial distance r from the center of the crypt; and longitudinal z distance from the top of the crypt downwards). Fig 4 illustrates this as a heat map plot for TFV concentration at 5 min after application of an enema to a small-sized crypt. The crypt fluid is “open”, i.e. contiguous with the fluid in the enema. The colors correspond to concentration values from 0 ng/mL (blue) to 10^9^ ng/mL (maroon). Due to the higher TFV diffusion coefficient in the fluid, crypt concentration within it is much higher than concentration in the surrounding tissue. The highest concentration in the tissue is found in the epithelium near the surface in contact with the enema fluid. In this plot, for clarity, only 20% of the length of the crypt is shown. The lower 80% contains much lower TFV concentration at the 5 minutes post dosing time illustrated here.

### Effects of variable enema retention time

**Fig 5:**
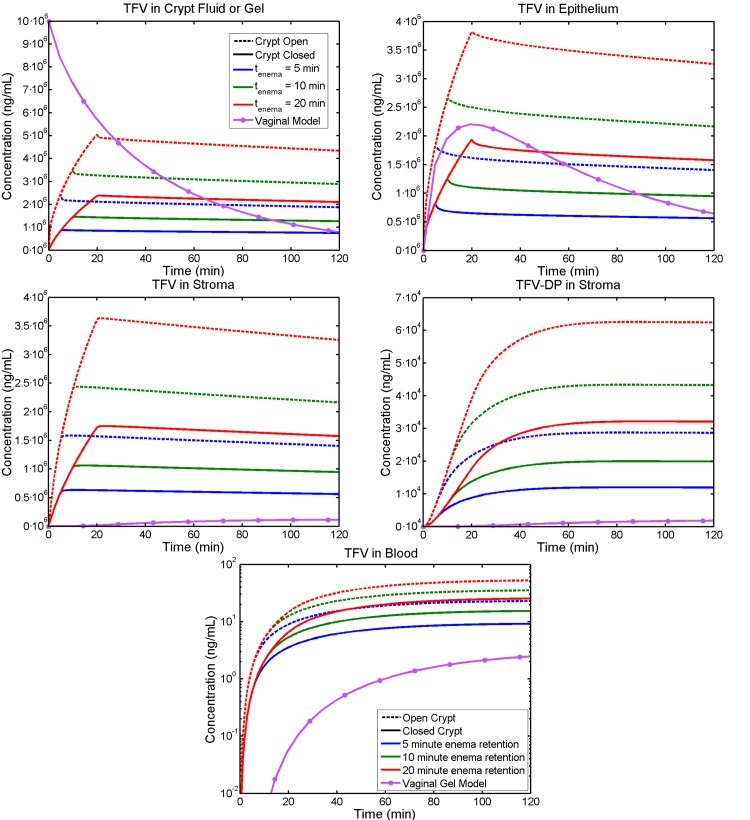
Volume-averaged TFV and TFV-DP concentrations vs. time after enema application in different compartments. Enema retention times are 5, 10, or 20 minutes (blue, green and red lines). A small sized crypt is contrasted between being open (hatched line) or closed to the luminal enema fluid (see further details in text). Results are also shown for compartmental volume-averaged concentrations vs. time, as computed for the earlier vaginal model for a tenofovir gel[20]; here there is also complete vehicle coating along the canal. Both vehicles initially contain 10^7^ ng/mL (1%) TFV. Note the much lower and slower developing concentrations in the vaginal mucosal tissue. Contrasts in rectal stromal TFV and TFV-DP concentrations for open vs. closed crypts are also evident.

Enema retention time is clearly a critical factor in drug delivery here, and we contrasted retention times of 5, 10 and 20 minutes. We assumed complete expulsion at the end of retention time (although that can be varied in the model). Results for times up to two hours post enema application are given in Fig 5. For contrast, we also included results for TFV concentration in the counterpart vaginal drug delivery model[20]; the purpose was to illustrate consequences of the very different luminal and mucosal structures of the vagina and rectum, and the sustained retention time of the gel. The subplots in Fig 5 are fluid in the crypt (or gel in the vaginal comparison), epithelium, stroma and bloodstream, and tenofovir diphosphate in the stroma. The crypt is taken as either open or closed.

TFV transport throughout the rectal epithelium is relatively rapid after enema application, and its concentration levels off soon after enema expulsion. The lag time to achieve maximum average TFV-DP concentration is noticeably longer than that for TFV. This is because the volume average includes lower levels in the stroma (deep into the crypt) to which TFV requires longer times to reach before being phosphorylated to TFV-DP. However, levels of TFV-DP in the upper stroma peak nearly as quickly as do TFV levels. TFV concentration in the blood shows a similar initial increase as in the stroma; but this increase continues after the stromal level is at maximum, because the drug continues to be taken up by the stromal vasculature faster than it is cleared by the kidneys. More TFV is delivered for an open crypt than for a closed one, since its transport rate within the space of the fluid filled crypt is much higher than that into the tissue, which has a lower diffusion coefficient. Increasing enema retention time markedly increases drug concentrations in all compartments.

### Effects of crypt size

**Fig 6:**
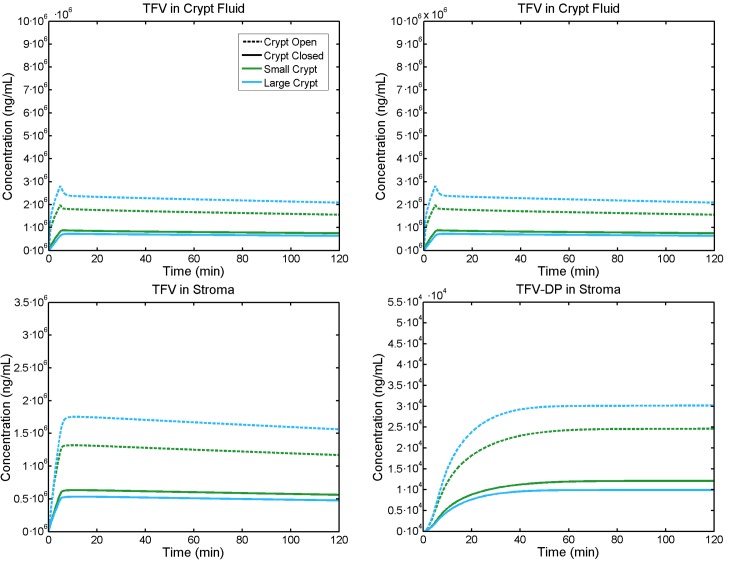
Volume-averaged TFV concentrations vs. time in different compartments. The lines indicate small (green lines) vs. large (blue lines) crypt sizes, which are open (hatched lines) or closed (solid lines). Enema retention time is 5 minutes. For closed crypts, size has a small effect. For open crypts, larger size increases concentrations by up to about 20%.

Results for a large versus a small crypt are shown in Fig 6. Again, the crypt interior has been taken as either open or closed to the enema fluid.

If the crypt is closed, the difference between a large and small crypt is minimal. This is because with a closed crypt, the primary mode of transport is through the epithelium at the surface, which has the same thickness for both crypt sizes. If the crypt is open, then the difference is much more pronounced, with a large crypt having higher TFV concentrations in the crypt fluid, epithelium and stroma, and higher stromal TFV-DP. The difference is approximately 50% for tenofovir in the tissue and approximately 25% for TFV-DP. Here, the concentration is actually lower for a large crypt for the closed case. This is likely due to the larger crypt leaching more tenofovir from tissue surrounding it.

### Effects of advective fluid flow within the crypt into the mucosal tissue

**Fig 7:**
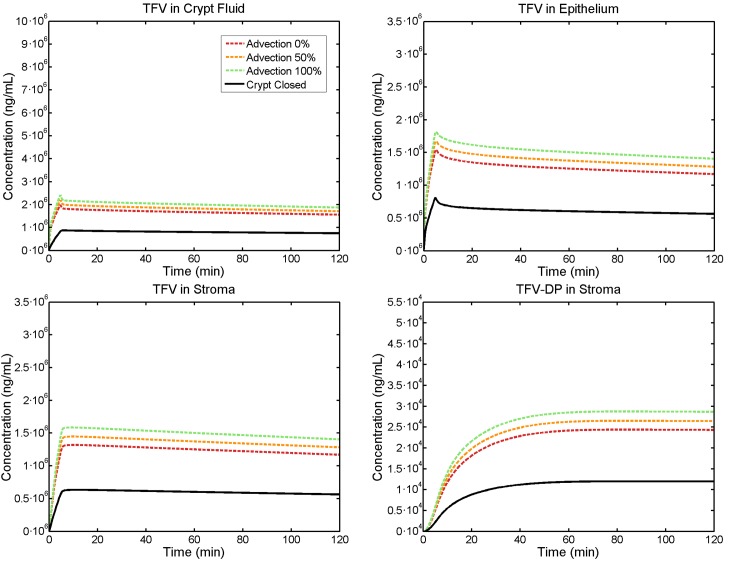
Volume average TFV concentrations vs. time in the compartments for varying advective flow. This is contrasted to a closed crypt (solid lines; see text for further details). Enema retention time is 5 minutes, and crypt size is small. Effects of advection on concentrations are relatively small (see text).

Fig 7 gives results for effects on drug transport of a maximum estimated advective velocity, one half its value, and no advection, again contrasting an open vs. closed crypt. Note that for a closed crypt, all advection is within the tissue, not in fluid within the lumen. For the open crypt the difference in stromal TFV-DP concentration between 0% and 100% maximum advective velocity is less than 20%. The effect here is small for fundamental physical reasons. API transport within a crypt is due to both diffusion and advection. Because the length scale of the crypt (0.1 cm) is so small, the drug transport rate by diffusion within that space is as high as that due to the maximum advective velocity (i.e. in fundamental terms, the Peclet number is of order unity).

### Detailed spatial-temporal tenofovir concentration profile

**Fig 8:**
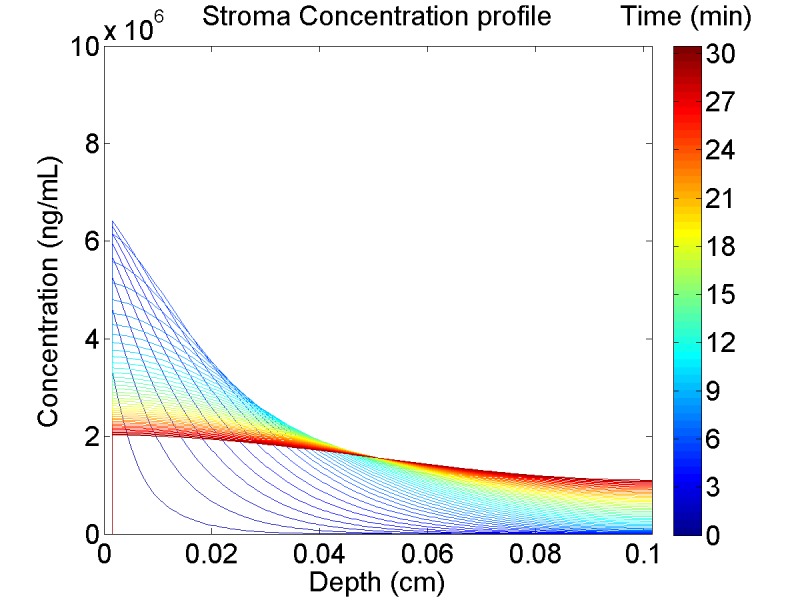
TFV concentration profile versus depth of the stroma. This is after 5 minutes of enema retention for a small, open crypt. Different times are indicated by different colors (cf. color bar), and the flattening of the concentration profile vs. depth with increasing time is clearly seen.

It is of interest to compute the local stromal drug concentration as a simultaneous function of position and time after enema insertion. This profile provides biophysical insight into the non-uniformity of TFV concentration distribution within the stroma, and consequently that of TFV-DP. For our vaginal PK modeling, accounting for this non-uniformity was consequential with respect to uncertainties in interpretations of concentrations in biopsies[20]. Fig 8 plots TFV concentration vs. depth down into the tissue at different times.

Here the concentration in the stroma at each depth is averaged in the radial direction for a particular time point (color axis, with blue for short and red for longer time points). The aspect ratio of the depth to the width varies from 8 to 18 depending on the size of the crypt. Because the radial stromal dimension (abutting the adjacent crypt) is small compared to the longitudinal size of the crypt downwards, the concentration variation in the former is small compared to that in the latter. The plot shows an initial high concentration near the surface of the tissue that gradually flattens out to the bottom of the crypt. A noticeable increase in concentration at the bottom of the stroma occurs around the 12 minute mark.

### Interpreting TFV-DP concentrations in stromal host cells–the “percent-protected” measure.

**Fig 9:**
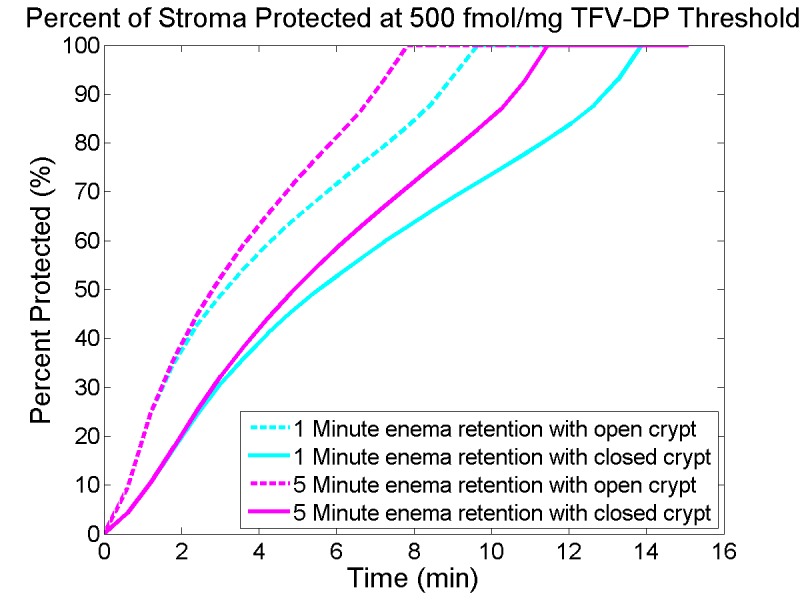
Percentage of stromal volume containing prophylactic TFV-DP concentration vs time. This is termed “percent protected” after 1 minute or 5 minutes of enema retention for a small crypt. See text for specification of “protected.” Contrasts between the shorter and longer retention times, as well as open vs. closed crypt status, are clearly seen.

The modeling approach here was previously used to create summary interpretations of TFV-DP concentrations in vaginal stromal host cells with respect to target values that are deemed prophylactic against HIV[11,12]. We conduct an analogous computation here, using as an example the target prophylactic TFV-DP concentration of 500 fmol/mg in tissue homogenate, i.e. a volume-average (which is an EC50 value)[32]. We compute that fraction of the entire stromal volume in which local TFV-DP concentration equals or exceeds this target value, as a function of time after enema application. This is termed “percent-protected.” Assuming uniform distribution of host cells, the percent-protected here (which is a tissue-based measure) is thus equal to the percent protected that could be based on a corresponding target prophylactic concentration in host cells per se. Notably, a human PK study including a 1% TFV rectal gel found good correlation between TFV-DP concentrations in rectal biopsy tissue homogenates and in isolated rectal mononuclear cells[33]. The time dependence of percent-protected after enema application is illustrated in Fig 9. The stromal volume is taken to extend 0.1 cm from the surface of the crypt. Below that distance, TFV and TFV-DP concentrations are negligibly low.

Here 100% percent protected is achieved with either 1 minute or 5 minutes of enema application. The time to 100% protection is longer for 1 minute vs. 5 minute enema retention times, and is also longer if the crypt is assumed to be closed.

### Simulating tenofovir and tenofovir diphosphate concentrations in biopsies

**Fig 10:**
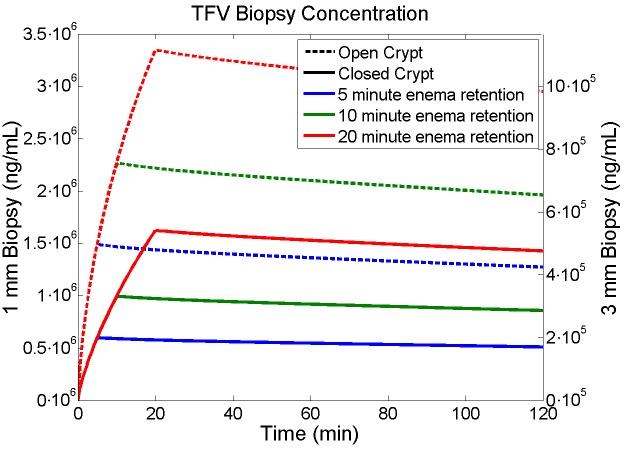
Results for TFV concentrations in a simulated biopsy. The biopsy contains both epithelium and some stromal tissue (see text). Enema retention time is 5 (blue lines), 10 (green lines) or 20 (red lines) min. Crypts are either open (hatched lines) or closed (solid lines). Biopsy thickness is either 1 mm (left y-axis) or 3 mm (right y-axis), and crypt size is small.

**Fig 11:**
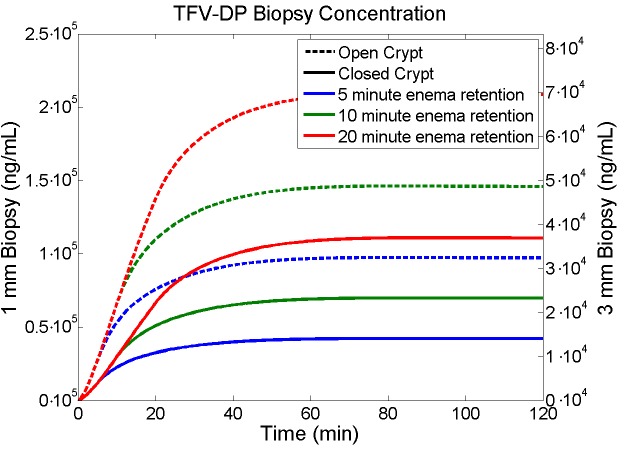
Results for TFV-DP concentrations in a simulated biopsy. The biopsy contains both epithelium and some stromal tissue (see text). Enema retention time is 5 min (blue lines), 10 (green lines) or 20 (red lines) min. Crypts are either open (hatched lines) or closed (solid lines). Biopsy thickness is either 1 mm (left y-axis) or 3 mm (right y-axis), and crypt size is small.

The detailed spatial-temporal mapping of concentrations of TFV and TFV-DP output by this model can be translated to simulations of concentration values in rectal biopsies, taken over varying times after enema application[20]. The nominal human rectal biopsy thickness is about 1 mm, but in practice can be thicker. Such a difference in thickness will influence the drug concentration typically measured as a mass average for the biopsy specimen. The model can be used to take a volume (de facto mass) average of concentrations in a geometric domain that simulates the tissue volume taken in a biopsy. Since the diameter of a crypt is about 40–120 μm and the diameter of rectal biopsy in a human is about two orders of magnitude larger, a single biopsy will sample a great many crypts. Thus, the concentration measured in a single crypt and in the tissue equidistant between it and adjacent crypts will represent concentration in the entire biopsy. Fig 10 and Fig 11 illustrate TFV and TFV-DP concentrations in a simulated biopsy for which the depth of the biopsy (i.e. tissue thickness in the volume average) is varied, and enema retention time and crypt openness are also varied.

**Fig 12:**
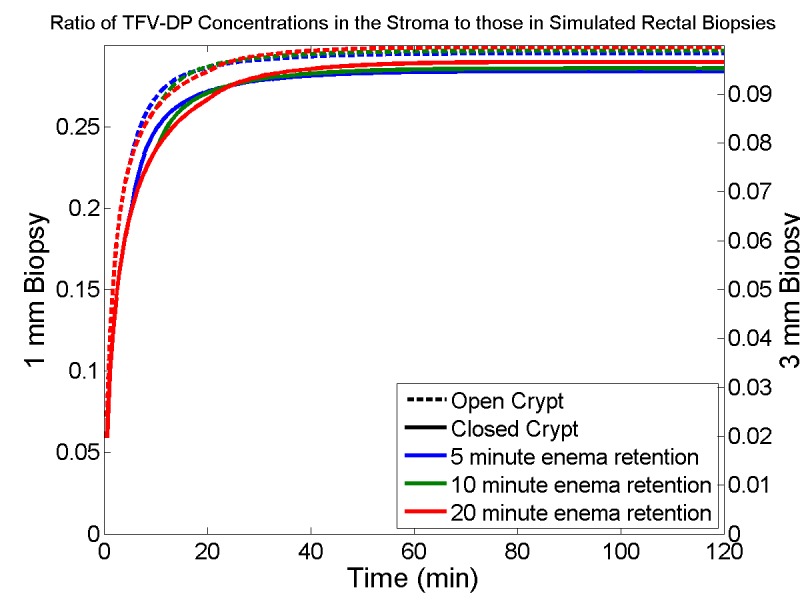
Ratios of predicted TFV-DP concentrations in the mucosal stroma to concentrations in simulated biopsies. The depth of the biopsy is assumed to be either 1mm or 3mm. Enema retention time is 5 min (blue line), 10 min (green lines) or 20 min (red lines). Crypts are open (hatched lines) or closed (solid lines). Notably, values in biopsies overestimate stromal values by about 3X (shallower 1 mm biopsy) to 9X (deeper 3 mm biopsy). Enema retention time and crypt openness have relatively small effects on this ratio.

Fig 10 and Fig 11 can be compared with Fig 5, for TFV and TFV-DP concentrations in individual compartments. The shapes of the two types of curves are qualitatively similar because there is so little drug that has diffused more than 1 mm into the stroma during the 2 hour time interval. Concentrations of TFV in 1 mm thick biopsies are similar to their values in both epithelium and stroma, which are also similar (Fig 10). Within 3 mm thick biopsies, TFV concentrations in biopsies are about one third those in the epithelium and stroma (Fig 10). TFV-DP concentrations in 1 mm biopsies are about 3X those in the stroma; if biopsy thickness is 3 mm, then this ratio increases to about 9X. Fig 12 gives the ratios of TFV-concentrations in stroma vs. biopsies.

## Discussion

Less is known about the functionality of rectal microbicides than about that for vaginal microbicides, and the overall effort on rectal microbicides is less than that for vaginal ones. Still, the epidemiological urgency to develop rectal microbicides is profound. Design and performance evaluation of vaginal and rectal microbicide products can benefit from deterministic compartmental PK modeling. The present work introduces a model for an enema vehicle that delivers tenofovir, to contribute to this effort. Such modeling can inform development of the rectal enema product by relating design-controllable characteristics of the enema (e.g. volume, rheology, drug loading) and its API (e.g. diffusion and partition coefficients, other physicochemical properties), as well as user-related factors (e.g. enema retention time), to measures of target PK. It can also help interpret drug concentrations measured in biopsies, and can inform sampling protocols for experimental PK studies. The detailed spatio-temporal characterization of drug concentration distributions within and between compartments and simulated biopsies provides objective predictions of when concentration maxima are achieved (t_max_ values), the magnitudes of those maxima (C_max_ values), drug concentrations at other specified times (e.g. C_24_ values), net drug delivered in specified times (e.g. AUC values), and quantitative predictions of how those values for biopsies relate to those for bioactive concentrations of drugs where they act against HIV (e.g. values in the stroma and its host cells). The results here are intended to demonstrate this capability as applied to the tenofovir enema.

The model here is a first step in computational modeling of rectal microbicide delivery, much akin to an initial one for vaginal delivery of tenofovir[20]. Here, as in the initial vaginal model, complete distribution of drug vehicle over the mucosal surface being analyzed is assumed, as discussed above in the Introduction section. The logical follow up is to incorporate the history of vehicle spreading, as we undertook for vaginal tenofovir gels[12], and plan to do here. This is more computationally demanding, and requires information about the kinetics and mechanics of vehicle distribution along the canal. Here, the low viscosity of the enema fluid suggests that it will spread relatively rapidly along the rectal canal (particularly if rinsed of feces by a prior cleansing enema), so that the time scale for such spreading is short compared to the longer time scales associated with mass transport of drug out from the enema fluid and into the mucosal tissue. Thus, the model here applies to that portion of the rectal canal over which the enema fluid has spread. Mass transport of tenofovir into the environs of a single, archetypal crypt is analyzed. Results depict concentration across that region, and can be related to those experimentally measured in a biopsy. Distributions of crypt sizes and spacings, and open vs. closed status, can also be readily incorporated into follow up modeling. In the absence of prior rectal cleansing and fecal washout, enema spreading and drug delivery will be less homogenous in space (along the canal) and in time (in relation to enema application and expulsion). Future modeling of this more complex process will be informed by imaging studies of vehicle distribution along the rectum[21,34].

Results here indicate four factors that could affect delivery of tenofovir by an enema: (1) duration of enema retention; (2) crypt status as open or closed; (3) crypt size; and (4) magnitude of advective flow from the lumen into the mucosal tissue. As seen in Fig 5 and Fig 9, enema retention time has a significant effect on TFV and TFV-DP concentrations. For example, doubling the time from 5 to 10 minutes causes almost a two-fold increase in stromal TFV-DP concentration (Fig 5). Decreasing retention time from 5 minutes to 1 minute increases the time to 100% protection from about 8 to 10 minutes for an open crypt, and from about 11.5 to 14 minutes for a closed crypt. These latter results also illustrate the significance of crypt openness, which doubles the peak stromal TFV-DP concentration per enema retention time. We emphasize that the contrast between open and closed crypts has physiological rationale, but that definitive characterization of it does not fully exist. Crypt size has a minor effect if the crypt is closed, but leads to about a 50% increase in stromal TFV-DP concentration if the crypt is open (Fig 6). As seen in Fig 7, advective fluid velocity (as estimated here based on rectal fluid absorption measurements) has a small effect on mucosal TFV and TFV-DP concentrations. This velocity decreases rapidly with distance from its maximum value in the apical portion of the crypt. Further, most of the early (e.g. < 30 min) drug transport from crypt fluid into the mucosa occurs in the upper crypt, so diffusion distances are small and diffusive transport is rapid. If higher values of advective velocity were experimentally obtained, they can readily be incorporated into this model.

Fig 10, Fig 11 and Fig 12 depict simulated TFV and TFV-DP concentrations in biopsies, illustrating contrasts for different biopsy thicknesses. In practice biopsy values of TFV are typically above the limit of experimental quantification (LOQ; usually measured with LC MS/MS). TFV-DP concentrations are typically much lower, and not always above the LOQ. It is TFV-DP concentrations in the stroma that are of greatest pharmacological relevance. These relate directly to concentrations in the stromal host cells which are infectible by HIV, and in which the reverse transcriptase inhibiting activity of TFV-DP can prevent viral replication. Biopsy thickness, in relation to the depth of the crypt, matters here with respect to comparing the volume averaged measures of TFV and especially TFV-DP concentrations vs. those in epithelium and stroma; however, the time courses of the changes in these concentrations are not appreciably different for the contrasting biopsy thicknesses. If stromal depth and biopsy thickness are comparable (1 mm in the example here) then TFV concentrations in biopsies are similar to those in the epithelium and stroma, which are also similar. However, if the thickness of the biopsy is greater than the depth of the crypt (3 mm thick in example here), then biopsy concentrations of TFV are reduced by about two thirds, and thus are lower than those in epithelium and stroma. For TFV-DP the situation is somewhat different. Here, for 1 mm biopsies, their TFV-DP concentrations are about three times those in the stroma. A threefold increase in biopsy thickness thus leads to its ninefold overestimation of concentrations in the stroma. If biopsy thickness were measured, then values in biopsies could be corrected to infer stromal values more accurately.

Results from this model should be compared with clinical data, but there are currently no published results for tenofovir PK after application of an enema. The closest data are from Yang et al[33] who studied effects of application of 4 mL of a 1% rectal tenofovir gel, with multiple dosing protocols. Gel distribution along the rectal canal, and consequent drug delivery, are likely to be different in time and space from those of an enema. That distinction notwithstanding, comparison of predictions here with the PK data for the rectal TFV gel can be instructive. Changing the units for TFV concentration in the Yang et al paper (ng/mg) to those here (ng/mL), the results for median biopsy concentration at 30 minutes are 0.581 X 10^4^ ng/mL, with an upper bound of 9.51 X 10^4^ ng/mL. In our model, predicted TFV concentration in a biopsy 30 minutes after enema application is of the order of 10^6^ ng/ml, ranging over a factor of about 10 depending upon enema retention time, and crypt openness (Fig 10). Thus, there is a difference of 1–2 logs with the gel data. Predicted values from the model for an enema are greater than measured values for 4 mL of gel with the same initial 1% tenofovir concentration. This difference could be due to a number of factors. The extent of the rectal canal covered by the gel, to the degree limited (by the high gel viscosity and presence of feces), could lead to reduced concentrations in biopsies taken in regions with little gel coating, because tenofovir transport within mucosal tissue longitudinally along the length of the canal is very slow compared to transport down into the layers of the mucosal tissue[20]. In contrast our model focuses upon a rectal segment along which there is complete enema vehicle distribution (longitudinal concentration distribution is not, yet, considered. Variations in thicknesses of the experimental biopsies and limited penetration of the TFV gel down into the rectal crypts (because of relatively high gel viscosity) could also contribute to these differences. As noted above, increased biopsy thickness reduces measured TFV concentration (e.g. average concentration in a 3 mm thick biopsy is about 1/3 of that for 1 mm biopsy in this model). Lack of the TFV gel penetration into crypts would compare to closed crypt status as defined here, reducing concentration by about a factor of two (Fig 10). Thus, relatively large biopsy thickness and poor, if not absent, gel penetration into crypts could cause as much as a 1 log reduction in TFV concentration in biopsies, the limitations of poor gel spreading along the rectal canal notwithstanding. Thus, differences between the predictions here for a TFV enema and the experimental PK data for a rectal TFV gel are not unreasonably different. We also note that the measured values of TFV concentration in rectal biopsies are much lower than those measured in vaginal biopsies after application of the same 4 mL volume of the same gel[35], and that predictions from our prior model agree well with vaginal PK data [20].

## Conclusions

We have introduced a deterministic model to apply in helping understand the multiple factors that govern drug delivery to the rectal mucosa and its sampling by biopsies, with application to a leading microbicide drug, tenofovir, as delivered by an enema. The model accounts for the geometrically distinct structure of the rectal mucosa, which gives rise to predictions of much faster rectal tenofovir delivery than that in the vagina. Examples have been presented for representative values of parameters characterizing rectal mucosal morphometry, tenofovir transport, and use-related factors such as enema retention time. These can be varied, to formally evaluate the sensitivity of model predictions to such variation[16]. Further, this sort of parameter sensitivity analysis can contribute to the understanding of population variability in experimental PK studies. The enema microbicide product has significant promise because: (1) the drug-delivering fluid will spread relatively rapidly along the rectal canal; (2) the modeling predicts that tenofovir enters rectal mucosal tissue quite rapidly (and tenofovir diphosphate has a very long half-life in host cells); and (3) precoital use of a microbicide enema would not alter behavior of a significant proportion of people who engage in receptive anal intercourse (who already use a cleansing enema precoitally). In follow up, the methodological approach introduced here can extend to other rectal dosage forms, as we have undertaken for vaginal microbicide products[12,20,36], and other microbicide drugs. Its predictions and contrasts can inform our fundamental understanding of rectal drug delivery, its experimental measurement, and the development of products and selection of their dosage regimens, as part of a rational product design process.
